# Pulmonary Susceptibility of Neonates to Respiratory Syncytial Virus Infection: A Problem of Innate Immunity?

**DOI:** 10.1155/2017/8734504

**Published:** 2017-11-09

**Authors:** Carole Drajac, Daphné Laubreton, Sabine Riffault, Delphyne Descamps

**Affiliations:** VIM, INRA, Université Paris-Saclay, Jouy-en-Josas, France

## Abstract

Human respiratory syncytial virus (RSV) is a common and highly contagious viral agent responsible for acute lower respiratory infection in infants. This pathology characterized by mucus hypersecretion and a disturbed T cell immune response is one of the major causes of infant hospitalization for severe bronchiolitis. Although different risk factors are associated with acute RSV bronchiolitis, the immunological factors contributing to the susceptibility of RSV infection in infants are not clearly elucidated. Epidemiological studies have established that the age at initial infection plays a central role in the severity of the disease. Thus, neonatal susceptibility is intrinsically linked to the immunological characteristics of the young pulmonary mucosa. Early life is a critical period for the lung development with the first expositions to external environmental stimuli and microbiota colonization. Furthermore, neonates display a lung immune system that profoundly differs to those from adults, with the predominance of type 2 immune cells. In this review, we discuss the latest information about the lung immune environment in the early period of life at a steady state and upon RSV infection and how we can modulate neonatal susceptibility to RSV infection.

## 1. Introduction

Human respiratory syncytial virus (RSV) was isolated for the first time in chimpanzees and identified in 1957 in children with severe lower respiratory illness [1–3]. RSV is an enveloped negative-sense single-stranded RNA (ssRNA) virus of the Pneumoviridae family [4]. RSV consists of a single serotype and two antigenic subtypes, A or B. The RSV genome is about 15 kb nucleotides and encodes nine structural proteins and two nonstructural proteins (NS1 and NS2). The envelope of RSV contains three transmembrane surface proteins, which are the fusion glycoprotein (F protein), the G glycoprotein (G protein), and the SH protein. F and G are the only RSV proteins that induce neutralizing antibodies [5].

RSV is the commonest viral agent causing acute lower respiratory infection (ALRI) in infants, a disease usually named bronchiolitis [6]. Bronchiolitis is characterized by mucus hypersecretion and inflammatory cell infiltration into the airspaces leading to airway lumen narrowing [7]. RSV infection is the main cause of hospitalization for severe bronchiolitis and is responsible for important infant mortality in developing countries [8]. RSV is highly contagious, and it is estimated that 95% of children have experienced at least one RSV infection before the age of two. Thus, the development of new treatment strategies is the World Health Organization's priority. A growing concern is that severe RSV infection may adversely affect pulmonary development and may lead to long-term respiratory disorders. Indeed, infants exposed to severe bronchiolitis or even to mild RSV disease are at much higher risk to develop recurrent wheeze up to teenage years [9].

In the sixties, the administration of formaldehyde-inactivated RSV vaccine (FIRSV) to a cohort of infants resulted in 80% of hospitalization due to an enhanced form of RSV-mediated disease (RSV disease). Two infants died and eighteen developed bronchiolitis and/or pneumonia, characterized by an excess of monocytes and eosinophils in the lungs [10]. High levels of anti-inflammatory type 2 T helper cell (T_H_2) cytokines, such as interleukin-4 (IL-4), IL-13, and IL-15, have also been detected in mice vaccinated with FIRSV [11, 12]. This dramatic episode highlights the need to improve our knowledge of infant immune responses to viral infection as well as of RSV pathogenesis in newborn airways. Although different risk factors (preterm birth, polymorphisms in host immune genes) are associated with acute RSV bronchiolitis [13, 14], the immunological factors contributing to the susceptibility of RSV infections in infants are not clearly elucidated. Different human epidemiological studies have established that the age at initial infection plays a key role in the susceptibility of RSV disease and the development of an asthma-like phenotype [15]. At birth, neonates, which rely on maternally derived antibodies (MDA) and innate responses, have a limited ability to defend themselves against pathogens. Indeed, the critical period of susceptibility to RSV arises between 2 and 6 months of age when MDA decrease beyond protective levels and before host neutralizing antibodies reach sufficient titers [16, 17]. Protection of preterm infants with higher occurrence of severe bronchiolitis (measured as the duration of wheezing) can be achieved through prophylactic treatment with a neutralizing humanized antibody called palivizumab [18]. Innate immune components that are the first available line of defense in neonates will also contribute to covering the “hole” in acquired immunity, educating the adaptive immune system, and strengthening it. Yet little is known about innate immunity in the lungs of neonates and how it will imprint further acquired immunity to RSV.

To better understand the immune pathways mobilized by RSV infection in infants and their long-term effects on the lungs, a mouse model of neonatal infection has been developed in BALB/c mice [19]. Mice infected under age 7 days (neonatal mice) develop an asthma-like pathology upon adult reinfection, characterized by weight loss, airway hyperresponsiveness, mucus hypersecretion, type 2 immune responses (neutrophil and eosinophil recruitment and IL-13 and IL-4 secretion), and airway remodelling [19, 20]. Thereby, as in human infants, the age of neonatal mice at initial RSV infection determines the clinical outcome upon RSV reexposure at adult age. These data suggest that RSV infection during the neonatal period is responsible for an immunopathological imprinting in the lungs that could influence the development and the severity of disease and finally long-term respiratory disorders. Thus, neonatal mice are an experimental model of interest to study the causes of this age-specific susceptibility.

The infant susceptibility to RSV infection is intrinsically linked to the immunological characteristics of the pulmonary mucosa. To date, neonatal innate immune responses and their effects on RSV disease progression remain poorly described. In this review, we resume the latest information about the immune environment in the young lung (mice and human). Then, the advanced researches on the mechanisms of the innate response to RSV infection in neonates are described. Finally, we discuss different approaches to modulate the young susceptibility to RSV infection by targeting the neonatal window of intervention.

## 2. Lung Tissue in Neonatal Life: A Moving Landscape

In mice, lung development begins at embryonic day 9 (ED9) and can be divided in three main periods, referred to as embryonic, fetal, and postnatal periods [21]. Before birth, lung development proceeds to pseudoglandular (ED12–16.5), canalicular (ED16.5–17.5), and saccular (ED18–PND4) stages forming the branching tree and future air space [21]. After birth, the formation of the secondary septa occurs resulting in the formation of the alveoli. This alveolarization phase takes place from postnatal day (PND) 4 to approximately PND21, with the development of the first mature alveoli and microvascular system around PND14 [21, 22]. During this period, the young lungs are exposed to a specific pattern of chemokines and cytokines, physical stress, and/or external environmental stimuli that will influence the immune system development [23, 24].

**Figure 1:**
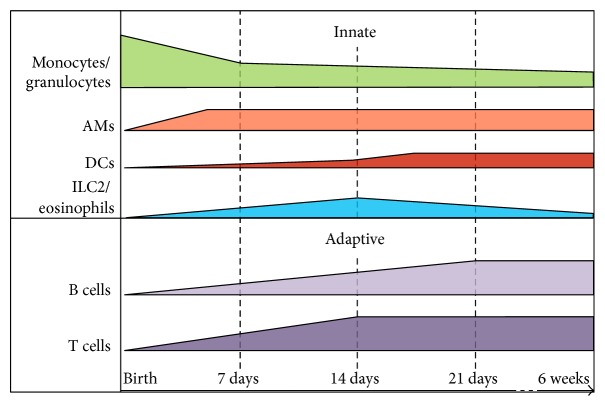
Immune cell colonization of the lungs during the postnatal period (schematization of cellular frequencies in CD45^+^ lung cells). Adapted from [25, 27, 30, 38] and personal unpublished data.

Immune cells start to colonize the lungs during the pseudoglandular phase (ED12) with the major population consisting of CD45^+^ yolk sac-derived macrophages, followed by fetal liver monocytes that enter the lungs at the beginning of the saccular phase (ED18) [25]. Following birth, lung epithelial cells produce elevated levels of GM-CSF (granulocyte-macrophage colony-stimulating factor or Csf-2). GM-CSF is a hematopoietic growth factor that promotes alveolar macrophage (AM) development in the lungs from fetal monocytes [25, 26]. AMs appear after PND1 in lung tissue and reach their maximum abundance in the lungs at PND3 where they fully colonize the alveolar space, coinciding with the start of the alveolar phase [25] (Figure 1). At PND14, monocytes, macrophages, and granulocytes reach adult-like cell frequencies [25, 27]. An influx of granulocytes (CD11b^+^ CD11c^int^ Ly6G/SiglecF^+^ cells probably corresponding to neutrophils) is quickly observed at PND1 [27]. Similar observations have been reported for circulating neutrophils. In human, following the first 24 hours after birth, the circulating neutrophil count abruptly raises and gradually stabilizes by 48 to 72 hours of life [28]. In neonatal mice, there is also a marked increase in circulating neutrophil numbers from PND1 to PND3 reaching an adult number from PND14 [29].

A recruitment of type 2 innate cells (type 2 innate lymphoid cells or ILC2s, mast cells, eosinophils, and basophils) occurs in the lungs at the start of the alveolarization period. Their frequency reaches a maximum at PND14 and then declines until weaning [27, 30]. A recent study showed that following the first breathes, the lung epithelium also produces high amount of IL-33 [30]. IL-33 is an alarmin that belongs to the IL-1 family and is mainly secreted by stromal cells such as epithelial and endothelial cells [31]. IL-33 signals through its receptor ST2 present in particular at the membrane of macrophages, dendritic cells (DCs), mast cells, and ILC2 [32]. IL-33 contributes to the promotion of T_H_2 immunity [31], particularly in the lungs of newborn mice [27, 30]. Indeed, IL-33 released by lung epithelial cells has been recently associated with the accumulation of ILC2 during the alveolar period [27, 30]. ILC2 cells belong to the family of innate lymphoid cells (ILCs). This family comprises three cellular groups that have been divided according to their cytokine production profile. Group 1 comprises both ILC1 and NK cells and is defined by the production of the signature cytokine IFN*γ*, while ILC2 has been defined by their ability to produce T_H_2-type cytokines such as IL-4 and IL-5 and ILC3 through their IL-17 production [33]. Thus, IL-33 secretion stimulates steady-state IL-5 and IL-13 production by ILC2 that in turn promotes a neonatal AM or DC phenotype switch towards type 2 immune response [27, 30]. To our knowledge, neither ILC1 nor ILC3 has been found in murine lungs [34, 35]. It is well known that NK cells represent up to 10% of resident lymphocytes in the lungs of adult mice [36], but their proportion in neonates has not been described yet.

GM-CSF also controls DC development in the lungs [37]. DCs are extremely rare in the respiratory tract after birth, but their frequency gradually increases over time. From E20 until PND2, CD11b^+^CD64^+^ monocyte-derived DCs (moDCs) form the majority of DCs. Our group showed that 6-day-old BALB/c neonatal lungs display less conventional (cDCs) and plasmacytoid DCs (pDCs), with a lower CD103^+^ to CD11b^+^ cDC ratio, as compared to adult lungs [38]. However, a study using a gating strategy that separates cDCs from moDCs in C57Bl/6J newborn mice showed that lung CD11b^+^ cDCs developed more slowly, leading to a predominance of CD103^+^ cDCs until PND7 [27]. During the alveolarization phase, neonatal DCs and pDCs display increased levels of OX40L (CD134) [27, 39] and this is related to their preferential ability to promote T_H_2 responses. Both IL-33 [27] and TSLP [39] production by lung epithelial cells seem to influence OX40-L expression in neonates.

As for DCs, T and B cells progressively accumulate in the lungs from birth to weaning [27]. Lung tissue of 6-day-old mice contain fourfold less CD3^+^ lymphocytes than adult tissue [38]. Neonatal T cell population is enriched in CD4^−^CD8^−^GATA3^+^ T cells while CD4^+^ and CD8^+^ T cells are less represented than those in in adults [38]. No difference in both NKT and *γδ* pulmonary cells are observed between adults and neonates. Little is known about B cells in neonatal lungs. At PND6, the neonatal lung tissues contain fivefold less CD19^+^ B cells than adult tissue [27]. Our group observed that the neonatal B cell population is enriched in both immature B cells and innate-like CD5^+^ B1a cells (Laubreton D. and Descamps D., unpublished data). A previous study has demonstrated that the CD5^+^ B cell population is more abundant in the spleen of 6-day-old C57Bl/6J neonates than in the adult spleen [40]. An equivalent subset named neonatal regulatory B cells (nBreg) has recently been identified in human cord blood [41].

First breaths not only provide signals that will shape lung maturation but also carry microbes that will form the microbiota. In neonatal mice, bacteria start to colonize the lungs around PND3, with their number and diversity progressively increasing until weaning [42, 43]. Interestingly, microbiota installation is closely related to the alveolarization phase. Indeed, Yun et al. suggest that bacteria influence lung development and barrier functions [44]. Bacteria can also influence the lung immune environment. In germ-free (GF) mice, invariant NKT accumulates in the lungs [45], and CD40 and programmed death-ligand 1 (PD-L1) expression by neonatal DCs is affected [42].

In conclusion, neonates display a lung immune system that is profoundly different from that of adults especially in regard to the presence of innate immune cells able to induce T_H_2 immunity. Moreover, the early life is a critical period for the lung development with the first expositions to external environmental stimuli and microbiota colonization. All these events affect the maturation of the pulmonary immune capacity and thus the lung susceptibility to respiratory pathogens [23, 24, 46].

## 3. Innate Sensing of RSV in Neonatal Lungs

At a steady state, the age-specific cellular composition of the neonatal lungs naturally promotes the initial development of T_H_2 immune responses [27, 30]. This ability can be influenced by innate responses of resident airway cells that produce different mediators following RSV sensing. Innate immune responses to RSV are important to control the early phase of viral infection but also to influence the polarization of anti-RSV immune responses and thus the outcome of RSV infection. The difficulty in studying lung cells in infants with bronchiolitis and in healthy controls leads to an incomplete knowledge of innate pulmonary immunity and factors influencing it in neonates. However, using animal models, numerous studies have identified that innate responses to RSV are decisive immunological events in neonatal RSV susceptibility [14, 47].

### 3.1. RSV Detection by Pattern Recognition Receptors (PRRs)

Several Toll-like receptors (TLRs), RIG-I-like receptors (RLRs), or nucleotide-binding domain and leucine-rich repeat-containing proteins (NLRs) are particularly involved in antiviral defenses and cytokine production upon RSV infection [14, 47]. Recognition of the virus by these PRRs is well defined in human and adult mouse but is still poorly described in neonates [48–50].

#### 3.1.1. Membrane TLRs Involved in RSV Recognition

The F fusion protein is recognized by TLR4, which is also known to detect lipopolysaccharide (LPS) of Gram-negative bacteria [51]. TLR4 stimulation leads to the production of proinflammatory cytokines and type I interferons (IFN-I), involving the signalling pathways dependent on two adaptive molecules which are the myeloid differentiation primary response 88 protein (MyD88) or the TIR domain-containing adapter inducing interferon *β* (TRIF). In TLR4-deficient C57BL10/ScCr mice, RSV clearance as well as activation and recruitment of NK cells is impaired [51, 52]. However, RSV infection is not affected in another TLR4-deficient BALB/c mice [53]. Human and murine pulmonary epithelial cells and macrophages express a broad range of TLRs including TLR4 [50, 54–56]. Interestingly, TLR4 signalling and IL-4R*α*/STAT6 and IFN-*β* pathway engagement in murine AMs promote a type 2 immune response in the course of RSV infection [57].

TLR2/TLR6 complex is also involved in the detection of RSV, but the mechanisms of sensing remain unknown. Activation of these receptors promotes the production of IFN-I and proinflammatory cytokines through the MyD88-dependent pathway [48–50]. In the lungs of TLR2- and TLR6-deficient adult mice, viral load is increased and neutrophil recruitment is impaired following RSV infection. Moreover, isolated AMs from these mice produce decreased levels of IFN-I and inflammatory cytokines [58].

The PRR expression by other mucosal innate immune cells like ILCs is better documented for the digestive tract than for the lungs [59]. Nevertheless, a recent work showed that TLR2 and TLR4 are expressed in pulmonary ILC2 and drive together a type 2 immune response by inducing IL-13 production [60].

#### 3.1.2. Endosomal TLRs Involved in RSV Recognition

TLR3 detects the double-stranded RNA form of the RSV genome, which is generated during the virus replication cycle [61]. TLR3 exclusively signals through the TRIF pathway. TRIF recruitment leads to the activation of the transcription factor interferon regulatory factor 3 (IRF-3), which generates IFN-I production by the cell. TLR3 is constitutively expressed in numerous cell types including nasal and pulmonary epithelial cells, AMs, and DCs [62, 63]. Rudd et al. showed that RSV promotes a type 2 immunity in TLR3-deficient adult mice with eosinophilic infiltration, mucus overproduction, and T_H_2-type cytokine secretion (IL-5, IL-8, and IL-13) while viral load remains unchanged [61].

TLR7 recognizes the single-stranded RNA genome of RSV and triggers subsequent expression of genes encoding IFN-I and proinflammatory cytokines via IRF-7 and NF-*κ*B activation through the MyD88-dependent pathway [48–50]. Lung epithelial cells, DCs, and eosinophils are able to sense RSV via TLR7 activation [56, 64–66]. RSV infection in TRL7-deficient mice induces a significant increase in inflammation and mucus production in the lungs [65]. Interestingly, Schlender et al. showed that RSV prevents IFN-I production in human pDCs in a TLR7-dependant manner, but they cannot explain the mechanism involved in such inhibition [67].

#### 3.1.3. Cytosolic Location of RLRs and NLRs

Retinoic acid-inducible gene I (RIG-I) and melanoma differentiation-associated gene 5 (MDA5) are both RNA helicases that can bind to the double-stranded RNA form of the RSV genome and 5′-triphosphorylated uncapped viral RNA in the cytosol [68, 69]. RIG-I signalling is particularly involved in IFN-I responses in lung epithelial cells, DCs, and AMs [56, 64, 68, 70]. RIG-I and MDA5 contain two N-terminal caspase activation and recruitment domains (CARDs), which, upon virus sensing in the cytosol, interact with the mitochondrial antiviral signalling (MAVS) protein to trigger the NF-*κ*B and IRF-3 pathways [48–50].

Nucleotide-binding oligomerization domain 2 (Nod2), a member of the NLR family, can also detect single-stranded viral RNA and triggers innate immune activation by binding with MAVS [71]. Upon RSV infection, MAVS-deficient mice displayed higher viral load in the lungs and profound defects in antiviral defenses in comparison with control WT mice, although RSV clearance is still effective in the absence of RIG-I, MDA5, and Nod signallings [64, 72]. Johansson's group showed that AMs are the main IFN-I producers through the MAVS-dependent pathway in adult lungs of RSV-infected mice [70, 73].

### 3.2. PRR Expression or Functionality, a Factor of Neonatal Susceptibility to RSV Infection

In infants, several genetic polymorphisms in innate immune genes have been associated with the susceptibility to develop RSV-mediated bronchiolitis [13, 74–76]. Thus, single-nucleotide polymorphisms (SNPs) in genes coding for PRRs have been considered attractive targets for clinical decision-making [77]. However, contradictory studies with other cohorts have failed to correlate SNPs in RIG-I or TLR4 genes with the severity of RSV disease [78]. Adult mouse models similarly show a variable role of the TLR4 pathway in the development of RSV disease [51–53]. Consequently, the severity of RSV disease appears likely dependent on both genetic and environmental factors (microbiota and coinfections) during the neonatal period [47]. Accordingly, a combination of the TLR4 genotype and environmental exposure to LPS during early life is involved in the occurrence of RSV bronchiolitis [79]. Most studies using cord blood cells suggest that TLR expression is not a modulator of the degree of cytokine responsiveness during the perinatal period [69, 80]. Furthermore, Marr et al. showed that RIG-I expression is similar between neonatal and adult pDCs [69]. They propose that IFN-I responses following RSV infection are decreased in neonatal pDCs compared to adult pDCs because of different signalling events downstream of MAVS or posttranslational modifications affecting either RIG-I or MAVS pathways [69].

Nevertheless, a correlation between pulmonary PRR acquisition after birth and RSV susceptibility cannot be excluded. Several studies in the mouse model showed that TLR4 expression is very low in the fetal lungs and increases throughout development [81, 82]. Harju et al. proposed an association between reduced pulmonary TLR4 expression at baseline and neonatal hyporesponsiveness to LPS [82]. Currently, there are virtually no studies on the maturation of TLR3 and 7 and RIG-I signalling in the neonatal lungs at a basal state and upon RSV infection.

### 3.3. Immediate Innate Responses of Pulmonary Resident Immune Cells to RSV Infection

**Figure 2:**
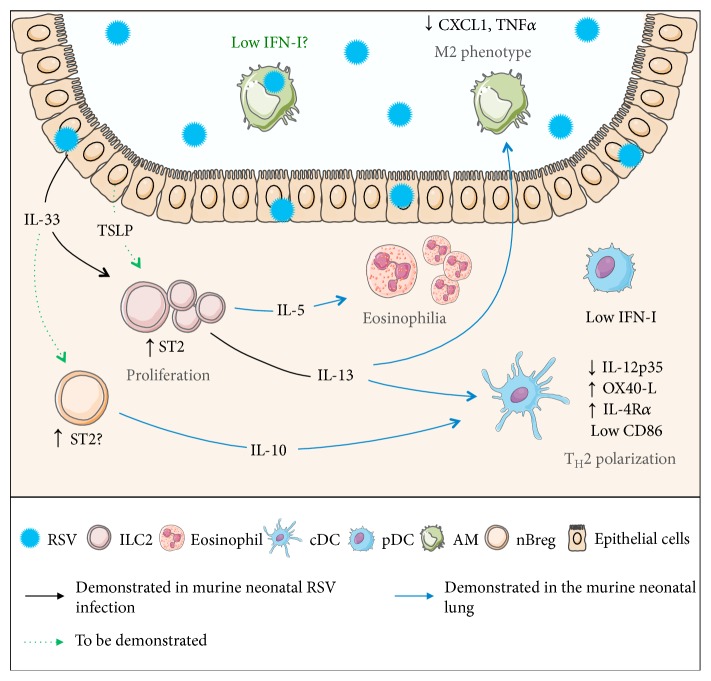
Immediate immune responses of pulmonary resident cells to RSV infection in neonates. Servier Medical Art has provided images. Neonatal RSV exposure leads to an early IL-33 secretion by respiratory epithelial cells [96]. IL-33 signals through its receptor ST2 localized at the membrane of ILC2. This alarmin supports the increase in the ILC2 number and IL-13 production in the lungs of RSV-infected neonatal mice [74]. ILC2 can promote a switch towards a type 2 phenotype for AMs or lung DCs at a steady state or in a house dust mite-induced asthma model [27, 30]. Concerning the IFN-I pathway, neonatal pDCs display a poor pulmonary mobilization and a weak activation of the IFN-I pathway following RSV infection [29]. AMs are the main source of IFN-I in RSV-infected adult lungs, but the question remains open during the neonatal period [54]. Therefore, it is strongly suspected that ILC2 cells are indirectly responsible for the inability of neonatal mice to mount an effective IFN-I response to counteract RSV infection. In addition, IL-10-secreting nBregs may constitute another cellular subset contributing to the type 2 immunity induced by RSV infection in neonates [40, 99].

RSV infection in neonatal mice promotes a type 2 immunity characterized by a strong proliferation of an IL-4R*α*^+^-CD4^+^ T_H_2 subset together with a defect in CD8^+^ T cell activation and IFN*γ* production [83, 84]. In this part, we describe the first innate responses of pulmonary resident cells to RSV infection that contribute to the development and/or maintenance of anti-RSV T_H_2 immunity (Figure 2).

Adaptive immune responses are initiated by DCs that traffic from the infected lungs to the draining respiratory lymph nodes in order to prime T cell responses. Our group and others have described major deficiencies in the functionality of DCs in neonatal lungs following RSV infection [85–87]. As compared to adults, cDCs are poorly represented in the lungs and in the lymph nodes of RSV-infected neonates, with an increased proportion of a CD103^+^ DC subset [85]. These neonatal DCs also have lower expression of the costimulatory molecule CD86 and thus are less effective in antigen presentation [85]. Moreover, a poor pulmonary mobilization of pDCs, potent producers of IFN*α*/*β*, and a weak activation of the IFN-I pathway are described in RSV-infected neonatal mice [86, 87]. IFN-I production is important not only to induce antiviral responses but also to amplify proinflammatory responses in the lungs of adult mice [88]. It has been demonstrated that IFN*α* treatment or an increased lung DC number (by adoptive transfer of adult pDCs or administration of a hematopoietic cell proliferation factor, the Flt3 ligand (Flt3-L)) reboots the IFN-I pathway upon RSV neonatal infection and decreases T_H_2-biased immunopathology upon adult reinfection [86, 87]. Thus, IFN-I production clearly appears as a key factor in neonatal susceptibility to RSV infection [86, 87, 89].

The role of AMs in primary RSV infection only begins to be appreciated. Thus, the depletion of AMs in the early period of life has been associated with a reduction in RSV clearance and a delay in weight gain [90]. In adult mice, AMs have also been described to play an essential role in early inflammatory molecule production (TNF*α*, IL-6, CCL3, and IFN*α*) and activation/recruitment of NK cells [91, 92]. Recently, it has been reported that adult AMs are the main source of IFN-I following RSV infection [70]. To date, these observations have not been checked in neonatal mice. AMs are known to be a flexible cellular subset that adapts to the microenvironment of the airway lumen [93]. It is not known yet whether neonatal AMs have the same reactivity to RSV infection than adult AMs. Addressing this issue seems critical to understand the causes of inability of a neonate to generate IFN-I response following RSV infection. Similar to the classification of T cells in a T_H_1/T_H_2 phenotype, macrophages have been also categorized into classically activated macrophages (CAMs or M1) or alternatively activated macrophages (AAMs or M2), based on activating cytokines (IFN*γ* and IL-4, resp.) and functional activities (inflammation and airway remodelling, resp.) [94]. Interestingly, Empey et al. have demonstrated that neonatal AMs present a delay of their differentiation toward a CAM phenotype following RSV infection, likely due to undetectable IFN*γ* production [95]. In adult mice, RSV infection induces AAMs that are important to reduce lung pathology [57]. Thus, pulmonary AM polarization seems to depend on age. Altogether, in the particular context of the neonatal airway environment, it becomes important to understand the mechanism that triggers the polarization of neonatal AMs following RSV infection and to evaluate its relationship with exacerbated airway responses upon adult reinfection.

The contribution of ILC2 and IL-33 to the neonatal RSV susceptibility has been recently investigated. Neonatal RSV exposure leads to an early IL-33 secretion by respiratory epithelial cells; this is not observed in adult mice. IL-33 plays a major function in the immunopathogenesis of RSV infection by supporting an increase in the ILC2 number and IL-13 production in the lungs of neonatal mice that will impact on disease severity in reinfected mice [96]. Additionally, it has been reported that hospitalized infants with viral bronchiolitis have detectable levels of nasal IL-13, IL-33, and thymic stromal lymphopoietin (TSLP) [96, 97]. Importantly, TSLP-deficient adult mice are unable to mount ILC2 proliferation and activation upon RSV infection [98]. The link between TSLP from respiratory epithelium and ILC2 proliferation/activation is not yet reported in RSV-infected neonatal mice. Nevertheless, the release of TSLP is identified as an important event for pulmonary DC polarization during RSV infection in the neonatal period and for RSV-mediated long-term respiratory disorders [39]. ILC2 is known to secrete T_H_2-type cytokines, such as IL-5 and IL-13. In the neonatal lungs, these cells can promote an AM or DC phenotype switch towards type 2-polarizing abilities at a steady state or in a house dust mite-induced asthma model [27, 30]. Therefore, it is strongly suspected that ILC2 cells are indirectly responsible for the inability of neonatal mice to mount an effective IFN-I response following RSV infection.

Finally, nBregs (or CD5^+^ B1a subset) may constitute another cellular subset contributing to the type 2 immunity induced by RSV infection in neonates. In the neonatal spleen, these nBregs have been previously described for their ability to produce IL-10 and to control the DC activation *in vivo* for T_H_1/T_H_2 polarization [40]. Moreover, IL-10-producing nBregs in the lungs could be induced by IL-33, as previously described in the intestines [99]. An equivalent subset of nBregs has been recently characterized in the blood of human neonates, and its frequency is identified as a predictive factor for the severity of RSV-mediated bronchiolitis in infants [41].

All these results point to the existence of T_H_2-like innate immune responses that are early induced by RSV infection in the neonatal lungs. These specific immunological properties must be considered in order to develop relevant therapeutic approaches against RSV infection.

## 4. Experimental Strategies to Modulate the Neonatal Susceptibility to RSV

An increasing set of data supports the concept of a “neonatal window of opportunity”. The early life is the critical period for the development of immunity and therefore for the newborn sensitivity to the development of pulmonary pathologies. Immunomodulatory interventions targeting this period of life are likely to have profound effects on immune system homeostasis and hence on an individual's susceptibility to pathogens [23]. Different studies using neonatal or adult mice have shown the possibility to take advantage of immunomodulation strategies on innate defenses to modify the neonatal pulmonary susceptibility to RSV infection and to fight RSV disease (Table 1).

### 4.1. Counteracting the Ineffective IFN-I Secretion in the Lungs

#### 4.1.1. Increase and/or Activation of IFN-I-Producing Cell Population

IFN-I production in neonatal RSV infection is decisive for the severity of RSV pathology [86, 87, 89]. Thus, with recombinant IFN-I intranasal instillation prior to mouse infection, Cormier et al. suggested that boosting the antiviral response of pDCs during the neonatal period limits RSV pathology [86]. Additionally, the treatment of neonatal mice with the Flt3-L, a growth factor for hematopoietic cells, before RSV infection increases pDCs in the lungs, partially restores the IFN-I pathway, and reduces the long-term pathological pulmonary consequences of RSV infection [87]. However, AMs have been identified as predominant producers of IFN-I in RSV-infected adult mice [70]. Therefore, the role of these cells in neonates and in the development of pulmonary anti-RSV immunity in the long term should not be ignored.

#### 4.1.2. Promoting IFN-I Signalling via TLR/RLR Agonists

By targeting PRRs, several groups propose the use of TLR or RLR agonists to boost antiviral responses. Thus, the preexposure of neonates to CpG (TLR9 ligand) prior to the first RSV infection reduces RSV pathology observed in the second RSV exposure at adult age. TLR9 stimulation induces the alteration of neonatal T_H_2 skewing, probably by accelerating maturation of neonatal antigen-presenting cells as well as NK cell recruitment in the lungs [100]. In an adult mouse model, others propose to target TLR3 with the synthetic dsRNA agonist poly IC stabilized with poly-L-lysine carboxymethyl cellulose (poly ICLC). However, the administration of poly ICLC fails to induce an appropriate innate immune response following RSV infection in the cotton rat model, which is not the case in BALB/c mice [101]. These results highlight the importance of the choice of an animal model used for therapeutic evaluation in the context of RSV infection. Recently, SB 9200, a dinucleotide prodrug targeting RIG-I and NOD2 activation, has been presented as a novel prophylactic and therapeutic anti-RSV immunomodulatory agent by Spring Bank Pharmaceuticals. In the mouse model, it has been observed that SB 9200 reduces viral load and lung inflammation while increasing IRF3-dependent IFN-I production [102]. To our knowledge, it is the first immunomodulation strategy targeting RLRs that could be considered in RSV treatment. However, because of the known dissimilarities in PRRs and cell immune responses according to age, it would be necessary to test SB 9200 in a neonatal model for assessing innate immune response following RSV infection.

### 4.2. Modulating the Activation of Cells Promoting the Pulmonary T_H_2 Environment in the Neonatal Period

#### 4.2.1. Modulating by Blocking T_H_2-Polarizing Cytokines

Several studies point out the major role of IL-13 in airway hyperresponsiveness of adult mice during RSV infection [20, 103]. Thus, IL-13 targeting could represent a good strategy to modulate neonatal responses to RSV infection. In adult mice, anti-IL-13 treatment prior to RSV infection reduces both viral load and mucus hypersecretion and increases IL-12 production in the lungs [103]. It would be interesting to study the effect of anti-IL-13 treatment on neonatal mice because it has been shown that IL-13 is highly secreted in the lungs upon neonatal RSV infection [96].

A recent study suggests that TSLP might also represent a therapeutic target for IL-13-driven immunopathology to RSV. Indeed, Stier et al. showed in adult mice that TSLP signalling is required for IL-13 production by ILC2. RSV-infected adult mice receiving an anti-TSLP neutralizing antibody presented a reduction in IL-13 production as well as a decrease in viral load and airway mucus secretion [98]. Furthermore, administration of anti-TSLP before neonatal RSV infection has been shown to reduce OX40-L expression on DCs thereby reducing their capacity to promote T_H_2 polarization and to decrease eosinophil numbers in the bronchoalveolar lavage fluids [39]. Both strategies (anti-IL-13 and anti-TSLP) are currently under clinical trial evaluation for adult patients with asthma [104, 105]. In the same way, the administration of an IL-33-neutralizing antibody during primary RSV infection in neonatal mice reduces IL-13 production and ILC2 numbers in the lungs and consequently decreases disease severity after reinfection at adult age [96].

#### 4.2.2. Modulating by Blocking Signalling Pathways Involved in the Type 2 Immunity Induction

Several groups propose to interfere with the T_H_2-biased immunopathogenesis of neonatal RSV infection by targeting receptors of T_H_2-type cytokines or proteins involved in their downstream signalling. Recently, Shrestha et al. have revealed that cDCs and pDCs downregulate their IL-4 receptor *α* (IL-4R*α*) with age. Interestingly, the elevated IL-4R*α* expression on CD11b^+^ cDCs is related to the immunopathology upon RSV reinfection [106]. Accordingly, the downregulation of pulmonary IL-4R*α* expression with antisense oligonucleotides (ASO) enhances the presence of maturation markers (CD80 and CD86) at the membrane of CD11b^+^ cDCs and leads to a shift of T cell responses toward T_H_1 cells producing IFN*γ*. Besides, IL-4R*α* ASO-treated neonates display higher level of T_H_1-like IgG2a antibodies in response to RSV exposure than nontreated mice, while viral load is unchanged. Subsequently, long-term respiratory disorders associated with RSV reinfection are reduced by the neonatal administration of IL-4R*α* ASO [107]. In accordance with these results, the inhibition of STAT6 activity, an essential transcription factor in IL-4R*α* signalling, by a specific inhibitory peptide during neonatal RSV infection, decreases IL-4 secretion and AAM number in the lungs and prevents from pulmonary eosinophil recruitment and airway hyperresponsiveness upon adult RSV reinfection [108].

#### 4.2.3. Modulating by Activation of Neonatal AMs

Several studies have reported that neonatal AMs present an immature phenotype upon RSV infection [90, 95] and this has been associated with T_H_2-biased airway immunopathology upon adult reinfection [108]. AM differentiation is controlled by local IL-4 or IFN*γ* secretion [94]. However, in neonatal mice, IFN*γ* production is absent following RSV infection [95]. Furthermore, it has been demonstrated that IFN*γ* production during neonatal infection influences the outcome of RSV pathology upon adult reinfection [109]. Indeed, it has been shown that intranasal injection of recombinant IFN*γ* in neonatal RSV-infected mice induces a better AM activation characterized by the expression of CAM markers (CD86^+^, MHC II^+^ and CCR7^+^, and mannose receptor^−^) on neonatal AMs and reduced viral load in the lungs [90, 95, 110].

### 4.3. Promoting the Maturation of the Pulmonary Immune System by Modulating the Microbiota

Several groups focused their research on the capacity of probiotic microorganisms to stimulate the lung immune system and to prevent RSV infection during the first years of life [111]. Studies were carried out with *Lactobacillus rhamnosus* isolated from goat milk in order to control RSV infection via TLR3 modulation [112–114]. Oral treatment of 3-week-old BALB/c mice with *L. rhamnosus* CRL1505 significantly reduces viral load and pulmonary tissue damage due to inflammation following RSV infection with respect to the control group [114]. Hence, *L. rhamnosus* CRL1505 administrated orally is able to beneficially modulate the respiratory mucosal immunity to RSV infection. The nasal administration of heat-killed probiotic *L. rhamnosus* CRL1505 is also able to increase the resistance of adult mice to the challenge with RSV [115]. Moreover, oral administration of this probiotic has reduced the frequency and severity of respiratory infections in a randomized clinical trial involving 298 children aged from two to five years in Argentina [112].

In parallel with the gut microbiota, a lung microbiota gradually colonizes the airways during the neonatal period [42, 43]. Its composition and diversity can affect host physiology and the immune capacity of the airway tissue [23, 111]. Thus, it has been observed that the nasopharyngeal microbiota of young children can influence the spread of RSV infection to the lower respiratory tract and can modulate the host immune response to virus [116, 117]. Thomas's group has recently isolated different primocolonizing bacterial strains of the mouse neonatal lungs in order to propose a new approach to modulate the immune response to respiratory pathologies. Thus, Remot et al. showed in a neonatal mouse model of asthma that the repeated administration of one strain of these primocolonizing lung bacteria positively or negatively impacts the outcome of pathology [43]. Therefore, it will be interesting to evaluate this strategy on RSV disease in neonates.

All these results indicate that the control of type 2 immune responses during primary RSV infection in the neonatal period can prevent RSV-mediated long-term respiratory problems. Altogether, the existence of the pulmonary neonatal environment displaying specific immunological properties must be considered in order to develop relevant therapeutic approaches against RSV infection.

## 5. Conclusion

In conclusion, the early response to RSV infection is closely associated with specific immunological characteristics of the developing lungs. That is why the RSV researches of new preventive or curative treatments against RSV must take advantage of experimental models in young animals. A better understanding of anti-RSV innate immune responses in neonates, and their relative contributions to long-term pulmonary immunopathology, is required to develop new immunomodulation—but also vaccination—strategies specific to this early period of life.

## Figures and Tables

**Table 1:** Biological therapeutic interventions to modulate neonatal innate immunity following RSV infection.

Strategy	Target	Design	Biological product	Category	Administration	Models	Ref.
Counteracting the ineffective IFN-I secretion	IFN-I-producing cells	Activation & recruitment	IFN-I and Flt3-L	rIFN-*α* and growth factor	Inhalation & injection	Mouse (N)	[86, 87]
	IFN-I signalling	Activation	CpG and SB 9200	TLR or RLR agonist	Oral	Mouse (A & N)	[100, 102]

Modulating the pulmonary T_H_2 bias	Th2-polarizing cytokines	Blocking	Anti-IL-13, anti-TSLP, anti-IL-33	Antibodies	Injection	Human (A) & mouse (A & N)	[39, 96, 98, 103–105]
	Signalling pathways	Blocking	Anti-IL-4R*α* and anti-STAT6	Antisense oligonucleotide and inhibitory peptide	Inhalation	Mouse (N)	[107, 108]
	Alveolar macrophages	Activation	IFN*γ*	rIFN*γ*	Inhalation	Mouse (N)	[90, 95, 110]

Modulating the mucosal microbiota	Respiratory & intestinal mucosa	Maturation	*Lactobacillus rhamnosus* CRL1505	Live or heat-killed bacteria	Oral or inhalation	Human (C) & mouse (N)	[112–115]
	Respiratory mucosa	Maturation	Primocolonizing lung bacteria strains	Live bacteria	Inhalation	Mouse (N)	[43]

A: adult; C: children; N: neonate.
